# Genomic and phenotypic insights point to diverse ecological strategies by facultative anaerobes obtained from subsurface coal seams

**DOI:** 10.1038/s41598-019-52846-7

**Published:** 2019-11-07

**Authors:** Silas H. W. Vick, Paul Greenfield, Sasha G. Tetu, David J. Midgley, Ian T. Paulsen

**Affiliations:** 1 Department of Molecular Sciences, Macquarie University, North Ryde, Australia; 2 Commonwealth Scientific and Industrial Research Organisation (CSIRO), Sydney, Australia

**Keywords:** Comparative genomics, Water microbiology

## Abstract

Microbes in subsurface coal seams are responsible for the conversion of the organic matter in coal to methane, resulting in vast reserves of coal seam gas. This process is important from both environmental and economic perspectives as coal seam gas is rapidly becoming a popular fuel source worldwide and is a less carbon intensive fuel than coal. Despite the importance of this process, little is known about the roles of individual bacterial taxa in the microbial communities carrying out this process. Of particular interest is the role of members of the genus *Pseudomonas*, a typically aerobic taxa which is ubiquitous in coal seam microbial communities worldwide and which has been shown to be abundant at early time points in studies of ecological succession on coal. The current study performed aerobic isolations of coal seam microbial taxa generating ten facultative anaerobic isolates from three coal seam formation waters across eastern Australia. Subsequent genomic sequencing and phenotypic analysis revealed a range of ecological strategies and roles for these facultative anaerobes in biomass recycling, suggesting that this group of organisms is involved in the degradation of accumulated biomass in coal seams, funnelling nutrients back into the microbial communities degrading coal to methane.

## Introduction

Globally, coal represents a key fuel, accounting for almost ~25% of the world’s energy consumption (International Energy Agency). The use of coal for power generation, however, is associated with significant environmental and health impacts^1,2^. As a cleaner alternative, methane derived from coal, known as coal seam gas (CSG) or coal bed methane has become an increasingly important ‘bridge’ fuel for a global transition to renewables. With the discovery that significant portions of the worlds CSG are produced through microbial degradation of the organic matter in coal to methane, has come an increasing interest in understanding the microbial communities involved in this process, with the aim of stimulating CSG production from coal reserves^3,4^.

Efforts to understand the process of biological coal degradation have focussed primarily on either (1) understanding what nutrients to add to stimulate microbial communities to degrade organic matter in coal to methane, and/or; (2) characterising the microbial community compositions on coal and associated formation waters either *in situ* on or in laboratory grown microcosms (reviewed in Ritter *et al*., 2015). These studies have typically used 16S rRNA gene based community profiling. This approach has been informative in characterising the members of coal-associated microbial communities, including which taxa appear to be ubiquitous in coal systems and those which appear to be dependent upon local conditions such as coal rank, depth and temperature. This type of research has enabled some generalisations to be made about the composition of these communities: all contain ubiquitous methanogenic archaea, a small number of very abundant bacterial taxa from the Proteobactera or Actinobacteria; and less frequently Firmicutes, and a long tail of rare organisms from a range of phyla^3,5,6^.

One key limitation of this approach is that assigned functions of any these uncultured organisms are obtained from related, cultured taxa. While this is useful for some groups such as the methanogenic archaea, where metabolic capabilities are largely constrained (see also^7,8^), it is considerably less reliable in the majority of bacterial groups where metabolic functions are not always taxonomically conserved. In order to address this, some effort to assign functions to coal seam microbes has been attempted through genome reconstructions from metagenomic sequences^9–11^. This approach has generated some insights into the metabolisms of members of these environments, notably the discovery of a novel methanogenic taxon^10^, however, as many genes in the process are unknown the effectiveness of this method is limited. Finally, classical isolation into either axenic or gnotobiotic culture can be used to elucidate the metabolic roles of individual organisms. Unlike metagenomics-based approaches, isolation has the advantage of being able to experimentally test the metabolic and physiological characteristics of the isolated taxa through growth studies, though a key drawback remains that many microbial species are recalcitrant to isolation.

In 2016, Vick *et al*. identified the presence of several early coal-colonists in a Sydney Basin formation water. One of the highly abundant OTUs (Operational Taxonomic Unit) observed in this 16S rRNA amplicon sequence based study was taxonomically identified as a pseudomonad (OTU_9), and its presence during early colonisation suggested it may play some role in degrading organic matter in coal. Interestingly, *Pseudomonas* species have been previously identified from almost all surveys of coal seams conducted to date^6,12–25^ and pseudomonads isolated from coals in the past have been reported for the production of biosurfactants^26^ and lignin degradation phenotypes^27^. Additionally, a previous study has noted the prevalence of seemingly aerobic taxa and metabolisms in deep anoxic hydrocarbon environments^28^. In order to try to assign functions to these coal-associated pseudomonads, and functionally related facultative aerobic taxa, a culturing effort was undertaken to bring facultatively aerobic organisms, including that corresponding to OTU_9, into axenic culture. This isolation was followed by genomic and phenotypic characterisation to uncover the metabolic and ecological roles played by these organisms in coal seams. As these organisms are facultative aerobes, the present study employed oxic conditions and a complex medium to obtain axenic cultures.

## Results

### Cellular and colony morphology

**Figure 1:**
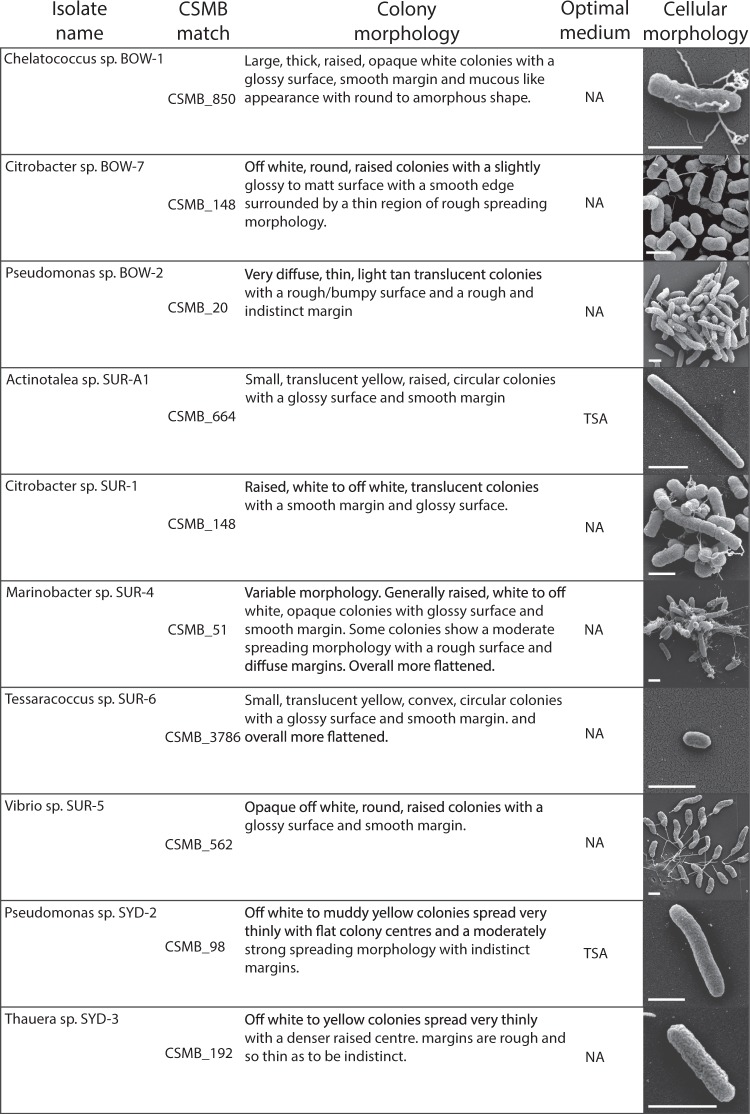
Colony and cellular morphology of isolates. Optimal growth media are nutrient agar (NA) and tryptone soy agar (TSA). Scale bars represent 1 µm.

Ten bacterial isolates were obtained from three coal enrichment cultures derived from coal seam formation waters from the Sydney, Bowen and Surat coal basins of eastern Australia. The isolates all grew under oxic and anoxic conditions on either NA (Nutrient agar) or TSA (tryptone soy agar). Cellular and colony morphology were characterised for aerobically grown cells on rich complex media (NA and TSA) and are summarised in Fig. 1. Taxonomy of isolates was determined with isolates found to represent members of the Alphaproteobacteria, Betaproteobacteria, Gammaproteobacteria and Actinobacteria (Fig. 1).

### 16S rRNA Phylogenetic analysis

**Figure 2:**
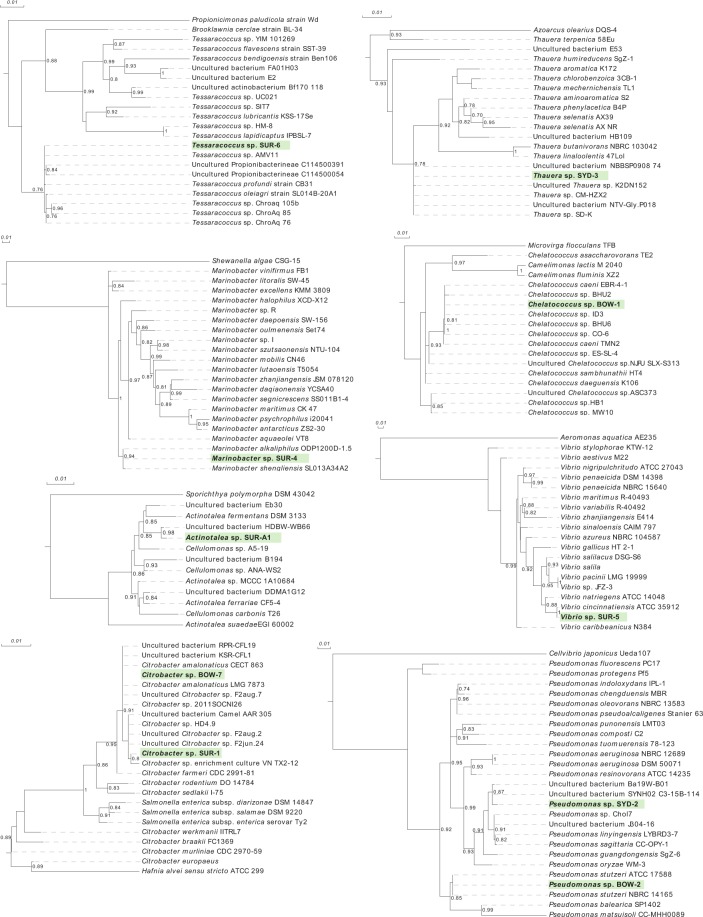
Phylogenetic tree analysis of coal seam isolates based on full length 16S rRNA gene sequences. Isolates from the current study are shown with bold font and highlighted in green. Numbers at branch junctions are a confidence index supporting branches as described in^67^.

Phylogenetic analysis of coal seam isolates was performed using the full length 16S rRNA gene sequence. Isolates are compared to previously characterised/reported members of each represented genera (Fig. 2). Analysis showed that *Chelatococcus* sp. BOW-1 is closely related to *Chelatacoccus caeni*, first isolated from a sludge biofilm wastewater reactor^29^. Both *Citrobacter* sp. BOW-7 and *Citrobacter* sp. SUR-1 isolates had highly similar 16S rRNA sequences and shared *Citrobacter amalonaticus* as their closest relative in the phylogenetic tree. *Pseudomonas* BOW-2 and *Pseudomonas* SYD-2 clustered within different species clades within the genus *Pseudomonas* tree. Bow-2 clustered within the *P. stutzeri* species clade, whereas SYD-2 clustered with a clade containing *P. linyingensis* and *P. sagittaria*. *Actinotalea* sp. SUR-A1, isolated from the Surat coal basin, showed the closest relationship to *A. fermentans*, a cellulolytic organism isolated from a municiple waste dumping site^30^. *Marinobacter* sp. SUR-4 sits in a clade shared by close taxonomic relatives *M. alkaliphilus* and *M. shengliensis*, isolated from alkaline sub-seafloor mud and oil contaminated soil, respectively^31,32^. *Vibrio* sp. SUR-5 has a very similar 16S rRNA sequence to *V. cincinnatiensis* and clusters closely to it in the *Vibrio* genus tree. *Tessaracoccus* sp. SUR-6 clusters within a clade containing *T. profundi* and *T. oleiagri*, previously isolated from the deep terrestrial subsurface^33^ and oil contaminated soil, respectively^34^. *Thauera* sp. SYD-3, isolated from formation waters sourced from the Sydney coal basin, does not cluster closely with any named species of *Thauera* but has very similar or identical 16S rRNA gene sequences to several unnamed environmental sequences from diverse industrial wastewater sources (Fig. 2).

Comparing isolate 16S rRNA sequences to those in the CSMB (Coal Seam MicroBiome) dataset^6^ identified the CSMB OTUs within which these isolates would be grouped. From this analysis *Chelatococcus sp*. BOW-1 matched CSMB_850, *Pseudomonas sp*. BOW-2 matched CSMB_20, *Pseudomonas sp*. SYD-2 matched CSMB_98, *Citrobacter sp*. BOW-7 and *Citrobacter sp*. SUR-1 both matched CSMB_148, *Actinotalea sp*. SUR-A1 matched CSMB_664, *Marinobacter sp*. SUR-4 matched CSMB_51, *Vibrio sp*. SUR-5 matched CSMB_562 and *Thauera sp*. SYD-3 matched CSMB_192.

### Biolog carbon utilisation phenotyping

**Figure 3:**
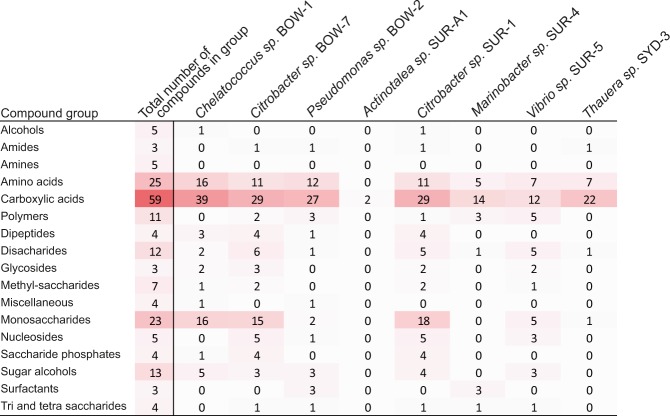
Heatmap showing the number of carbon substrates supporting growth in the various compound categories as measured by reduction of a tetrazolium dye on Biolog phenotype microarray plates 1 and 2. Isolates *Tessaracoccus* SUR-6 and *Pseudomonas* SYD-2 are not included as they appeared unable to grow under the conditions supplied by the Biolog assay.

Biolog phenotype microarray plates PM1 and PM2 were used to characterise the catabolic capabilities of the coal seam bacterial isolates under aerobic metabolisms. Two strains, *Tessaracoccus* SUR-6 and *Pseudomonas* SYD-2, showed no observable growth or respiration in Biolog media under the conditions required for the assays, and were therefore not able to be assessed for carbon utilisation profiles. Other isolates grew on a range of different carbon substrates with the exception of *Actinotalea* sp. SUR-A1, which only showed growth on the carbolic acids: acetic acid and acetoacetic acid (Fig. 3).

### Ecophysiological genome analyses

In order to better understand the lifestyles and metabolic strategies of the bacterial taxa isolated in the current study from coal seam formation waters, the genomes were examined for genes and genetic elements relevant to ecological lifestyle. Isolates were all isolated under oxic conditions indicating they are capable of using oxygen as a terminal electron acceptor and the requisite metabolic pathways/enzymes for this are observed in each genome. The genomes of both *Citrobacter* isolates (BOW-7 and SUR-1) and *Actinotalea* SUR-A1 encoded genes for the respiratory nitrate reductase (NarG) indicating that they are capable of using nitrate as a terminal electron acceptor also. *Actinotalea* SUR-A1 was also predicted to be able to use sulphate as a terminal electron acceptor. IMG/ER annotation also revealed that *Pseudomonas* BOW-2 was the sole isolate to carry the genes necessary for nitrogen fixation.

### Aromatic hydrocarbon degradation

**Figure 4:**
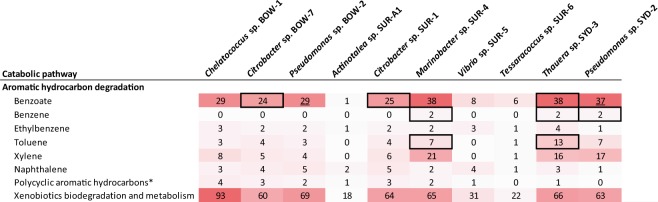
Number of genes in catabolic pathways for aromatic hydrocarbon degradation as determined by KEGG pathways analysis. Counts in enclosed boxes indicate a complete catabolic path and underlined counts indicate pathways containing all but one enzyme in the pathway. *With the exception of naphthalene.

Isolate genomes were also analysed with KEGG (Kyoto Encyclopedia of Genes and Genomes) to determine the presence of aromatic hydrocarbon degradation genes and pathways. All isolates except *Actinotalea* SUR-A1, *Vibrio* SUR-5 and *Tessaracoccus* SUR-6 showed a high number of genes in the benzoate catabolism category, a central intermediate in several aromatic degradation pathways. This included three complete pathways and two pathways missing only a single gene component. Additionally, *Marinobacter* SUR-4 and *Thauera* SYD-3 contained complete catabolism pathways for benzene and toluene, while *Pseudomonas* SYD-2 contained complete pathways for benzene only. Interestingly, all benzene catabolism pathways observed appeared to proceed through the intermediate catechol, indicative of aerobic catabolism rather than benzoyl-CoA which is indicative of anaerobic catabolism (Fig. 4).

### Carbohydrate-active enzymes

**Figure 5:**
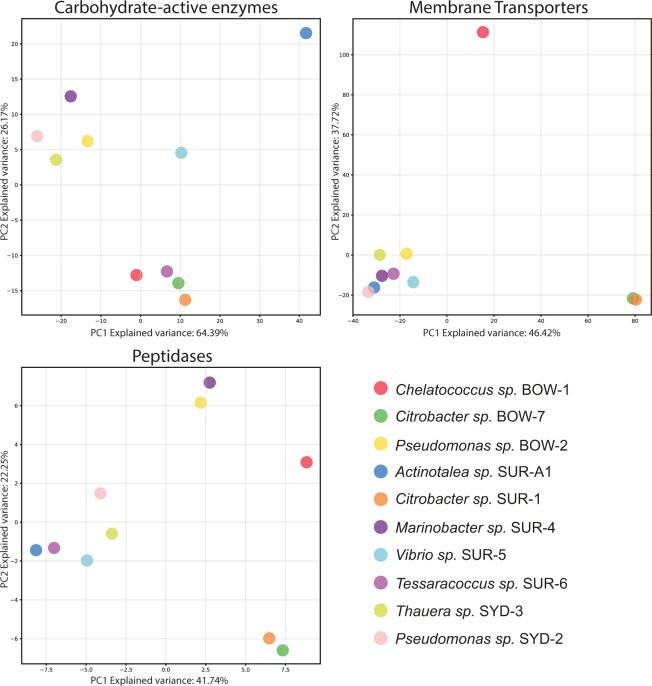
Principle components analysis (PCA) of gene abundances from genome annotations of carbohydrate-active enzyme, membrane transporter and peptidase genes. Abundances were measured as gene counts for the different classes of carbohydrate-active enzyme and peptidase genes (Online Resource 1) and gene counts for the different membrane transporter gene classes (or predicted substrates for ABC transporters) (Online Resource 1).

Predicted protein sequences from all isolate genomes were annotated for carbohydrate-active enzyme genes using dbCAN to assess the capabilities of isolates for utilising various carbohydrates^35^. Genes which matched carbohydrate-active enzymes were then analysed with signalP to determine if the enzymes were likely excreted or transferred to the outer cell surface^36^. A number of carbohydrate-active enzyme genes were identified in all of the isolate genomes (Supplementary Material 1). PCA (Principal Component Analysis) of these carbohydrate-active enzyme gene profiles identified two main clades containing similar profiles. These consisted of *Pseudomonas* BOW-2, *Pseudomonas* SYD-2, *Thauera* SYD-3 and *Marinobacter* SUR-4 in the first group, characterised by having relatively fewer glycoside hydrolase (GH) and glycosyl transferase (GT) genes, and *Chelatococcus* BOW-1, *Tessaracoccus* SUR-6, *Citrobacter* BOW-7 and *Citrobacter* SUR-1 in the second, which had higher numbers of GH and GT genes. Outside of these two clusters *Vibrio* SUR-5 and *Actinotalea* SUR-A1 were outliers, having markedly different carbohydrate-active enzyme gene profiles (Fig. 5). SignalP analysis of the carbohydrate-active enzyme genes in the isolate genomes revealed that *Actinotalea* SUR-A1 encodes significant numbers of carbohydrate binding module (CBM), GH and S-layer homology domain (SLH) genes with accompanying signal peptide sequences, suggesting that this isolate can form a cellulosome. *Vibrio* SUR-5 also appeared to have a high number of GH and CBM genes, however it lacked the SLH genes characteristic of cellulosomes and a lower number of its genes contained signal peptides (Supplementary Material 1).

### Membrane transporters

Genomes were annotated using TransportDB 2.0 to identify genes involved in membrane transport as these genes provide information on metabolism, through import transporters, and resistances to certain toxic compounds through export transporters^37^. Isolate genomes contained between 263 and 850 genes involved in membrane transport from 90 transporter families (Supplementary Material 1).

PCA of the transporter profiles in the ten isolates showed that most isolates had a similar membrane transport gene profile while *Chelatococcus* BOW-1 and both *Citrobacter* isolates (BOW-7 and SUR-1) were outliers having quite different profiles (Fig. 5). The difference in the *Chelatococcus* BOW-1 membrane transporter profile appeared to be driven by a number factors including: a larger number of membrane transport genes overall, a higher number of TRAP-T (Tripartite ATP-independent Periplasmic Transporters) family of transporters, involved in dicarboxylate uptake, and a larger number of ABC transporters involved in the uptake of amino acids, sulphate and polyamines. Differences in the *Citrobacter* transporter profiles appear to be driven by increased numbers of sugar transporters in the SSPTS (Sugar Specific Phosphotransferase System), GPH (Glycoside-pentoside-hexuronide) and ABC transporter families, and amino acid transporters in the HAAAP (Hydroxy/Aromatic Amino Acid Permease) and APC (amino acid-polyamine-organocation) transporter families (Supplementary Material 1).

### Peptidases

Analysis using the MEROPS database identified a suite of putative peptidase genes in all isolate genomes^38^. PCA of the peptidase gene family profiles for the isolates identified a group of isolates with similar peptidase profiles. This group, which included *Pseudomonas* SYD-2, *Thauera* SYD-3, *Vibrio* SUR-5, *Tessaracoccus* SUR-6 and *Actinotalea* SUR-A1, was characterised by a lower total number of putative peptidase genes. Other genomes with higher peptidase numbers separated out, included *Marinobacter* SUR-4 and *Pseudomonas* BOW-2 which group together, *Citrobacter* BOW-7 and SUR-1 also grouping and *Chelatococcus* BOW-1, with the highest number of peptidase genes, sitting as a outlier (Fig. 5) (Supplementary Material 1).

### Secondary metabolites

**Figure 6:**
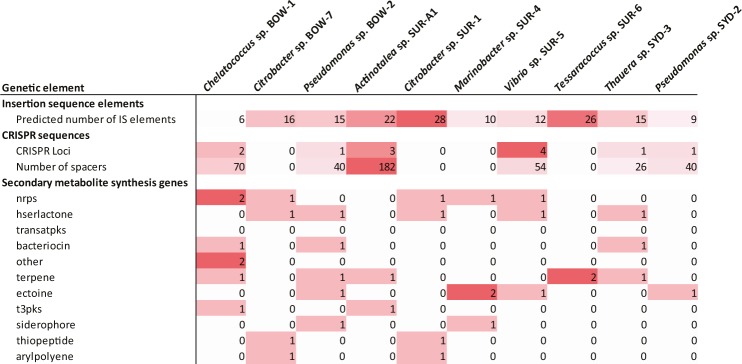
Heatmap showing the abundance of genes or genetic elements observed in the genomes of coal isolates. nrps (non-ribosomal peptide synthetase), hserlactone (homoserine lactone), transatpks (trans-AT polyketide synthase), t3pks (type 3 polyketide synthase).

The antiSMASH tool was used to annotate secondary metabolite gene clusters from the isolate genomes, of interest due to the roles secondary metabolites play in bacterial competition and biosurfactant production for substrate solubilisation^39^. All isolates showed the presence of secondary metabolite gene clusters from a range of classes including non-ribosomal peptide synthetases (nrps), homoserine lactones (hserlactone) commonly used for quorum sensing, bacteriocins involved in competitive bacteriocidal interactions, ectoine used as a compatible solute and siderophores used for iron scavenging (Fig. 6).

### Insertion sequences and CRISPRs

In addition to genes produced by bacterial species for catabolism and competition the presence and abundance of insertion sequences and CRISPRs (clustered regularly interspaced short palindromic repeats) were investigated to assess horizontal gene transfer and viral predation on the bacterial isolates. Analysis using the ISsaga tool identified numerous insertion sequence elements in all isolate genomes with *Citrobacter* SUR-1 and *Tessaracoccus* SUR-6 having notably high numbers while *Chelatococcus* BOW-1 had notably less than other genomes (Fig. 6)^40^. Genome analysis with CRISPRFinder (https://crispr.i2bc.paris-saclay.fr/Server/) identified CRISPR sequences in six of the isolate genomes while four didn’t appear to have any CRISPR elements (Fig. 6)^41^. It is noteworthy that the size of CRISPR spacer arrays was quite variable between isolates while the number of CRISPR loci was less variable (Fig. 6).

## Discussion

*Pseudomonas* represents a ubiquitous bacterial genus observed in almost all surveyed coal seam microbial communities. Despite this, the ecological and metabolic roles played by these pseudomonads and related facultatively aerobic taxa in coal seams remains poorly understood. The current study aimed to isolate representatives of these pseudomonads from multiple coal seam environments and to characterise their capabilities both genomically and phenotypically, in order to better understand their roles in this environment. Aerobic isolation from three coal seam formation waters on peptide rich media successfully generated two pseudomonad isolates from different species clades. This isolation effort also resulted in the generation of eight additional bacterial isolates from the actinobacterial genera *Tessaracoccus* and *Actinotalea* and the proteobacterial genera *Thauera*, *Marinobacter*, *Chelatococcus*, *Citrobacter* and *Vibrio*. As a phylogenetically diverse range of bacterial isolates were obtained it was decided to pursue genomic and phenotypic characterisation of all bacterial isolates to investigate putative ecological functions for this set of facultatively anaerobic, heterotrophic bacteria. The cellular morphology of all isolates was examined through scanning electron microscopy, carbon metabolisms characterised through Biolog phenotype screening and genomes sequenced and annotated to examine ecophysiologically relevant genes and genetic elements.

The roles of microbes in coal seam microbial communities are typically thought of in terms of their carbon catabolism, where the organic components of coal are sequentially degraded to methane in a linear fashion by a succession of microbial species. The ecological functions in a microbial community, however, are more complex and include a range of community functions including primary and secondary degradation, predation, scavenging and biomass recycling, amongst others. In fields of macro-ecology these ecological roles are often interrogated in terms of life strategy categories such as the competitive, stress tolerant and ruderal (CSR) classification used to classify the ecological strategies of plants during succession^42^. These lifestyle classifications and the catabolisms and phenotypes associated with them are, in microbial systems, dependent upon the type of environment and how the energy and nutrient resources are introduced into the environment^43^. The coal seam constitutes an endogenous heterotrophic environment in that it is a closed system without regular microbial, nutrient or energy inputs from external sources^43^. In this type of environment initial colonising ruderal species are heterotrophic organisms which modify the physicochemistry of the environment through their metabolic processes until the environment is modified to a point where slower growing, stress tolerating organisms or those with specialised metabolic capabilities are able to compete and proliferate^43^.

**Figure 7:**
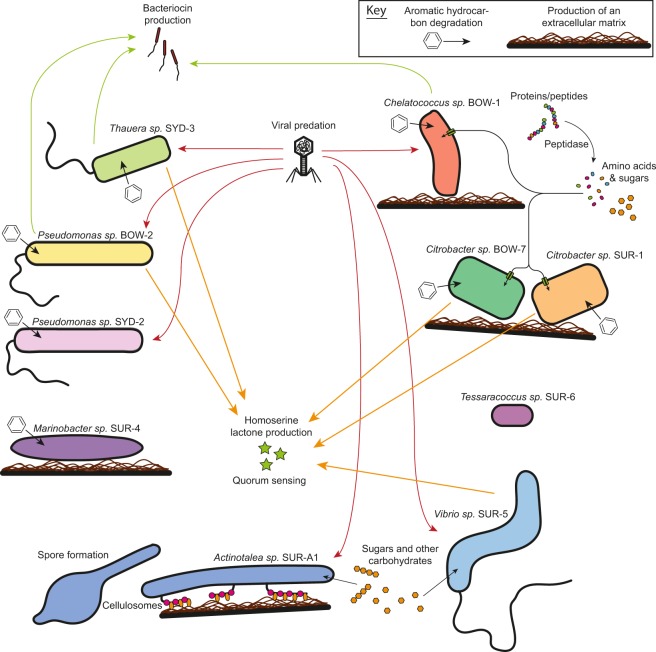
Conceptual model of the ecologically relevant capabilities of bacterial isolates determined through genomic and phenotypic analysis.

Examination of the genotypic and phenotypic analysis of isolate from the current study suggests the presence of three broad ecological life strategies. The first resembles a ruderal life strategy and is shared by *Citrobacter* SUR-1, *Citrobacter* BOW-7 and *Pseudomonas* BOW-2, characterised by rapid growth on common, labile biomolecules with few genes associated with specialised metabolisms. The second life strategy appeared to be an opportunotrophic lifestyle as described by Singer *et al*.^44^. This was characterised by genes associated with diverse metabolisms suitable for survival across a wide range of different environments and was observed in *Thauera* SYD-3, *Marinobacter* SUR-4 and *Pseudomonas* SYD-2. The third life strategy group represents the specialists. This group is broader in function and includes different specialist strategies but shares the common trait of having narrower metabolisms with a greater genomic investment in a targeted catabolic process. Isolates which could be considered to have specialist life strategies include *Chelatococcus* BOW-1 and *Actinotalea* SUR-A1. In addition to isolates that had characteristics aligning with known ecological strategies, two isolates (*Tessaracoccus* SUR-6 and *Vibrio* SUR-5) had life strategies that did not appear to conform to known models based on the genotypic and phenotypic characterisation performed here (Fig. 7). From our analyses of the isolates and their genomes described here, it is not believed that any of these isolates partakes in a lifestyle or strategies centred around direct biodegradation of coal compounds *in situ*.

The first life strategy group constituted isolates displaying a ruderal life strategy. Comparison of the isolates from the current study to OTUs observed in a previous study of succession in coal seams, using the CSMB OTU reference set^6^, shows that *Citrobacter* SUR-1, *Citrobacter* BOW-7 and *Pseudomonas* BOW-2 matched to OTUs which, in a previous study, showed initial rapid proliferation, quickly dominating the communities, before declining in abundance at later time-points^45^. This pattern of growth is characteristic of ruderals which rapidly proliferate on the most labile compounds available in an environment before being replaced by microbes with more specialised metabolisms once this labile resource is exhausted. In a previous study, these OTUs matching *Citrobacter* SUR-1, *Citrobacter* BOW-7 and *Pseudomonas* BOW-2 have also showed a very high level of variation in relative abundance across replicate microcosms compared to other taxa in the community. This high variation in abundance is due to competition by different ruderal taxa in other replicates as success as a ruderal is often decided by small stochastic affects during inoculation^46^. The phenotypic and genotypic features presented in the current study support this hypothesis of a ruderal lifestyle and suggest catabolic niches supporting them. These putatively ruderal isolates all showed growth on a wide variety of amino acids, peptides and carboxylic acids (Fig. 3). The genomes of these organisms also contained large numbers of peptidase genes and membrane transport genes for amino acids, sugars and carboxylic acid transporters (Supplementary Material 1). This collection of genes and phenotypes suggests that growth on cellular debris, particularly proteinaceous material, may drive the initial ruderal response in coal seam microbial communities. This cellular debris likely originates from cell death due to stress associated with the inoculation event but in native coal environments cellular debris could be liberated by chemical or physical perturbations to the coal seams or the ingress of meteoric waters.

The second life strategy type observed amongst our isolates was an opportunotrophic lifestyle, *sensu* Singer *et al*.^44^. Isolates conforming to this grouping (*Thauera* SYD-3, *Marinobacter* SUR-4 and *Pseudomonas* SYD-2) have been observed in a previous study of succession in coal seams^45^. In this previous study these opportunotrophic organisms remained at low abundances during the succession process^45^. These were also the only isolates to have considerable numbers of genes involved in aromatic hydrocarbon degradation (Fig. 4). Interestingly, in all of these organisms the pathways proceed through the metabolic intermediate catechol, an intermediate indicative of aerobic catabolism^47^. As all the eastern Australian coal seams sampled in the current study are highly anoxic and chemically reduced it is unlikely that these isolates are using these pathways for aromatics degradation in the coal seam under the conditions from which they were isolated. It should be noted, however, that these aromatic molecules are common in these coal seams and these may become available in the more oxic regions of aquifers into which coal seam formation waters may migrate. This observation of genes involved in aerobic aromatics catabolism, along with the facultative anaerobic growth patterns of these isolates, suggests that many of these organisms have diverse metabolic potentials allowing for persistence and growth across a large range of environments, scavenging diverse carbon compounds when environmental conditions allow it. A lifestyle in line with that described for the prototypical opportunotroph *Marinobacter aquaeolei*^44,48^. It is noteworthy that two pseudomonads with distinctly different metabolic and ecological lifestyles were observed in this study. This likely reflects the wide metabolic and lifestyle diversity in the genus which spans parasitism to mutualism^49–51^ and free-living lifestyles^52^.

Amongst the isolates to be aligned with the specialist life strategy group is *Chelatococcus* BOW-1. *Chelatococcus* BOW-1 displays very high number of diverse membrane transport genes, particularly ABC transporters involved in the uptake of amino acids, sugars, and polyamines. This factor, coupled with its growth on a wide range of carbon sources and high peptidase gene number but non-ruderal succession observed in previous studies and paucity of genes involved in aromatic hydrocarbon degradation suggests that this organism likely specialises in scavenging low quantities of diverse cellular material for nutrients and energy. This lifestyle would allow this organism to survive in diverse environments so long as other organisms were present to provide it with biomass to recycle. Similarly, *Actinotalea* SUR-A1 showed a distinctive ecophysiological profile setting it apart from the other isolates examined. *Actinotalea* SUR-A1, in contrast to *Chelatococcus* BOW-1 showed a reduced metabolic diversity compared to other isolates having a low number of transporters and growing on very few carbon compounds. *Actinotalea* SUR-A1 did, however, display a large number of carbohydrate active enzymes including all components involved in the formation of a cellulosome^53^. This suggests *Actinotalea* SUR-A1 may be responsible for complex polysaccharide degradation in the coal seam, presumably from biofilm or extra-cellular material as has been previously reported to be produced by coal seam microbial communities^24,45^. The genome of *Actinotalea* SUR-A1 also displayed a number of traits involved in cellular defence including a large number of CRISPR elements and a large number of daunorubicin and other multidrug efflux pumps often associated with antibiotic production in Actinobacteria^54^. Together with the observed production of spores by this isolate (Fig. 1), these results indicate that *Actinotalea* SUR-A1 follows a stress tolerator lifestyle, relying on a specialised metabolism based around carbohydrate catabolism rather than a broader opportunitrophic lifestyle, although sharing metabolism of common biomolecules as well as the ability for environmental persistence through sporulation.

A small number of isolates from the current study (*Tessaracoccus* SUR-6 and *Vibrio* SUR-5) did not appear to conform to any clear life strategy models based on the genotypic and phenotypic characterisations performed in the current study. It may be that these organisms are also involved in biomass recycling albeit on a smaller range of cellular biomolecules than the other scavenger and opportunotroph organisms. Alternatively, it may be that these organisms are involved in novel or unexamined catabolic niche processes in coal or other subsurface waters.

From a broader perspective, some notable differences were observed in the numbers of insertion sequence and CRISPR elements between the different isolate genomes. One interesting observation was the large difference in the number of insertion sequence elements observed in the closely related *Citrobacter* isolates BOW-7 and SUR-1 (Fig. 6). This increase in insertion sequence number in *Citrobacter* sp. SUR-1 may indicate recent adaptations as insertion sequences have been implicated in genomic shuffling and reorganisation^55^. CRISPR elements have previously been reported as unexpectedly common in a metagenomic exploration of a deep fractured shale environment and have been suggested to be a common feature of the terrestrial subsurface^56^. The current study supports these findings with observations of CRISPR sequences in six out of ten isolate genomes examined. If this high abundance of viral particles in subsurface environments is indeed the case, then viral predation may be a contributing factor in controlling biomass turnover, cell density and species distributions in subsurface coal environments, as it has been shown to be in other microbial environments^57^. This could have implications for subsequent methane production by methanogenic coal seam communities and warrants further attention as a potential limiting factor for methane production rates from coal.

Much of the literature in the field of coal seam microbiology is focused on improving coal seam gas production rates through nutrient and microbial amendments^3^. This has meant that studies have primarily focused on the microbial compositions of formation waters and coal solids, considering less the continuous nature of aquifers and whether those organisms that live within them require flexibility as they may find themselves in different physical and chemical environments along the course of the aquifer. Further, it would be valuable to be able to distinguish those taxa that are subsurface generalists from those that are coal seam specialists. A concept that has recently been explored by Barnhart and colleagues^58^. Based on their metabolisms as facultative anaerobes and their genomic potential for recycling of cellular biomass, it is likely that the isolates from the current study belong to a group of subsurface generalised organisms rather than coal seam specialists. It should be noted, however, that the current observations are made from genome sequences and expression of these genes under *in situ* conditions remains undetermined. Examination of expression of these genes under anaerobic conditions mimicking the coal seam environment may shed further light on the lifestyles of these microbes.

In summary, bacteria in coal seams are thought to carry out the majority of steps involved in coal degradation, with methanogenic archaea responsible for only the very final stages of conversion of acetate, CO_2_ or other simple methylated compounds to methane^3^. These initial stages of degradation, carried out by the bacteria taxa in the community, are thought to be the rate limiting step in the conversion of coal to methane and so identifying and characterising the bacterial taxa responsible for these processes is important for understanding the microbial coal to methane conversion process^4^. The current study reports on the successful isolation of two pseudomonads and eight other bacterial isolates, from eastern Australian coal seam formation waters. Phenotypic and genomic characterisation of these bacterial facultative anaerobes from the coal seam identified metabolisms supporting ruderal, opportunitrophic and specialist lifestyles centred around recycling carbohydrates and proteinaceous material in the coal seam. This study represents an initial step in assigning ecological and metabolic functions to the bacterial taxa observed in coal seams and utilised a protein rich isolation media and aerobic isolation conditions to target members of the genus *Pseudomonas*, which have been identified as ubiquitous members of coal seam microbial communities.

Future work should focus on isolation strategies to target other metabolisms involved in the conversion of organic matter in the coal to methane, these include anaerobic hydrocarbon degraders to identify taxa responsible for breakdown of hydrocarbons in coal or sulphate reducing bacteria which constitute a large proportion of coal seam microbial communities in eastern Australian coal seam formation waters despite the absence of elemental sulphur and sulphate from these environments and for whom a definitive catabolic role is not yet known.

## Methods

### Sources of isolates

Formation water for enrichment cultures was sampled anoxically from the wellhead of CSG wells in the Bowen Basin (QLD, Australia), Sydney Basin (NSW, Australia) and Surat Basin (QLD, Australia). 250 mg/L of Na_2_S, 200 mg/L of cysteine HCl and resazurin to a final concentration of 0.0001% were added to the formation water immediately upon collection. The formation water was transported to the laboratory and incubated anoxically on crushed coal from matching formations at 30 °C (Bowen and Sydney) and 40 °C (Surat) for several months prior to use as an inoculum. Chemical analysis of formation water was performed by the ALS Environmental (Sydney Australia).

Actively methanogenic enrichment cultures were generated from these Bowen, Sydney and Surat Basin formation waters. To mimic the coal seam environment as closely as possible sampled waters were used as a culture medium with 25 mg/L NH_4_Cl, 400 mg/L K_2_HPO_4_.2H_2_O added to increase growth rates. Gamma sterile (8 kGY) crushed coal (<1 mm) collected from the same coal basins as the corresponding formation waters was used as the sole carbon source. An anoxic headspace consisting of 95% Argon: 5% H_2_ was used in the butyl rubber stoppered vials and these enrichment cultures were incubated for several months at temperatures matching those measured in the produced formation waters (40 °C for the Bowen and Sydney Basin and 30 °C for the Surat Basin).

### Isolation of bacteria

In order to isolate facultative aerobic taxa, 50 µL of enrichment culture was plated onto multiple nutrient agar (NA) (Lab-lemco powder 1 g/L, yeast extract 2 g/L, peptone 5 g/L, NaCl 5 g/L and agar, 15 g/L) plates which were then incubated under oxic conditions at temperatures matching those of the corresponding enrichment cultures until colonies were apparent. Colonies were then picked based on morphological differences and streaked multiple times on NA and tryptone soy agar (TSA) (Pancreatic digest of casein 15 g/L, enzymatic digest of soya bean 5 g/L, NaCl 5 g/L and agar 15 g/L) plates to produce axenic cultures. Isolates were screened to identify novel isolates by colony PCR amplification of the 16 S rRNA gene using Bioline MyTaq following the manufacturer’s instructions. Amplicons were purified (QiaQuick PCR purification kit) and sequenced by the Ramaciotti Centre for Genomics in both the forward and reverse directions (University of New South Wales, Sydney, Australia).

### Visualisation of cellular morphology

To examine cell morphology isolates were grown in 10 ml of nutrient broth (same as for NA excluding agar) overnight at 40 °C for Bowen and Sydney basin isolates and 30 °C for Surat basin isolates with shaking (140 rpm) or until visible turbidity was observed. 200 µl of broth culture was then spotted onto poly-L-lysine (mol. wt. 150,000–300,000) coated glass coverslips for 5 min. Broth culture was then removed and the coverslips incubated in 3% glutaraldehyde in 0.01 M phosphate buffer (pH 7.4) for 24 hours at room temperature before being washed three times, each for 10 min with 0.01 M phosphate buffer. The coverslips were then incubated for 10 min in each of 20%, 50%, 70%, 80% and 90% ethanol solutions, twice with 100% ethanol before final dehydration with a Leica EM CPD300 critical point dryer. Coverslips were mounted on metal stubs, coated with 20 nm of gold with an Emitech K550 gold sputter coater unit and imaged using a JEOL JSM-7100F field emission scanning electron microscope operating at 5 kV accelerating voltage.

### DNA extraction and whole genome sequencing

Isolates were grown as for visualisation. Cells were pelleted by centrifugation and genomic DNA was isolated using either the Isolate II genomic DNA kit (Bioline), PowerSoil® DNA Isolation Kit (MO BIO Laboratories) or FastDNA® KIT (MP Biomedicals). Library preparation and whole genome sequencing were performed at the Ramaciotti Centre for Genomics (University of New South Wales, Sydney, Australia) using the Nextera XT sample preparation kit (Illumina) and an Illumina Miseq platform to generate 250 bp paired-end reads. Sequence reads were error corrected using Blue (http://bioinformatics.csiro.au/blue) before *de novo* assembly with Velvet 1.2.10^59,60^. Assemblies were submitted to the Genomes Online Database (GOLD) (https://gold.jgi.doe.gov/) and annotated using the IMG-ER pipeline^61^. Genome assemblies are available under the project accession ID’s: Gp0191703 (*Chelatococcus sp*. BOW-1), Gp0191702 (*Pseudomonas sp*. BOW-2), Gp0191704 (*Citrobacter sp*. BOW-7), Gp0191706 (*Actinotalea sp*. SUR-A1), Gp0151178 (*Citrobacter sp*. SUR-1), Gp0191707 (*Marinobacter sp*. SUR-4), Gp0191708 (*Vibrio sp*. SUR-5), Gp0191709 (*Tessaracoccus sp*. SUR-6), Gp0191711 (*Pseudomonas sp*. SYD-2) and Gp0151176 (*Thauera sp*. SYD-3).

### 16S rRNA phylogenetic analysis

16S rRNA gene sequences were taken from IMG annotations of genomes or through the use of the bioinformatic tool Kelpie (https://github.com/PaulGreenfieldOz/WorkingDogs/tree/master/Kelpie). BLAST searches were conducted against the NCBI RNA reference sequence database to find closest relatives and percentage identity. 16S rRNA gene sequences for representative sequenced relatives of each of the isolates were obtained from GenBank. Phylogenetic trees were constructed from 16S rRNA gene sequences using the default settings at the Phylogeny.fr webserver (http://www.phylogeny.fr.)^62^. In brief, sequences were aligned with Muscle^63^, the alignment checked and corrected using Gblocks^64^, the tree structure calculated using PhyML^65^, prior to visualisation using TreeDyn^66^. Branch support for the 16S rRNA phylogenetic tree was carried out using the aLRT method to bootstrap analysis^67^.

### Biogeography of isolates

In order to examine the biogeography of isolates, the V4 region of 16S rRNA gene sequences of isolates were matched to the Coal Seam MicroBiome (CSMB) OTU dataset, which represents a large set of environmental survey OTUs from studied coal seams worldwide^6^. Sequences were matched using the -usearch_global command from the USEARCH software with an identity threshold cut-off of 97.5%^68^. CSMB OTU matches were examined to determine the set of locations where the OTUs best matching each cultured strain had been observed previously (10.4225/08/5ab47c2bc8cfb)^6^.

### Ecophysiological genome analysis

Genomes were annotated for predicted coding sequences using the PROKKA software^69^ and predicted protein sequences functionally annotated using: CRISPRFinder^41^, ISsaga^40^, TransportDB 2.0^37^, dbCAN^35^, antiSMASH^39^, MEROPS^38^ and signalP 4.1^70^.

### Carbon utilization phenotyping

Carbon utilisation capabilities of isolates were assayed with the Biolog microplate system (Biolog, California). PM1 and PM2 (Phenotype Microplate) carbon source microplates were used according to the manufacturer’s instructions with either the gram negative or positive protocol depending on the gram stain of the isolate. Respiration was measured by colourimetric changes caused by the reduction of a tetrazolium redox indicator dye mix. The dye mixes used for the individual isolates were chosen according to manufacturer recommendations based on taxonomic affiliation and rates of growth of the isolates. Dye mix A was used for BOW-2, BOW-7, SUR-1 and SYD-3; dye mix H was used for BOW-1, SUR-A1, SUR-4, SUR-6 and SYD-3 and dye mix D was used for SUR-5. Inoculated Biolog microplates were incubated in the Omnilog automated incubator-reader for 36 hours at temperatures matching those found *in situ* for the coal seams from which isolates were obtained: 40 °C for isolates BOW-1, BOW-2 and BOW-7, 30 °C for isolates SUR-A1, SUR-4, SUR-5, SUR-6 and Sur-1 and at 37 °C for isolates SYD-2 and SYD -3 with absorbance at 590 nm measured at 15 minute intervals for each well.

## Supplementary information

Supplementary material 1
